# Flowing atmospheric pressure afterglow combined with laser ablation for direct analysis of compounds separated by thin-layer chromatography

**DOI:** 10.1007/s00216-015-9165-5

**Published:** 2015-11-12

**Authors:** Michał Cegłowski, Marek Smoluch, Edward Reszke, Jerzy Silberring, Grzegorz Schroeder

**Affiliations:** Faculty of Chemistry, Adam Mickiewicz University in Poznan, Umultowska 89b, 61-614 Poznań, Poland; Department of Biochemistry and Neurobiology, Faculty of Materials Science and Ceramics, AGH University of Science and Technology, Al. Mickiewicza 30, 30-059 Kraków, Poland; ERTEC-Poland, Rogowska 146/5, 54-440 Wroclaw, Poland; Center of Polymer and Carbon Materials, Polish Academy of Sciences, M. Skłodowskiej-Curie 34, 41-819 Zabrze, Poland

**Keywords:** Thin-layer chromatography, Mass spectrometry, Laser ablation, Flowing atmospheric pressure afterglow, Ambient ionization

## Abstract

A thin-layer chromatography-mass spectrometry (TLC-MS) setup for characterization of low molecular weight compounds separated on standard TLC plates has been constructed. This new approach successfully combines TLC separation, laser ablation, and ionization using flowing atmospheric pressure afterglow (FAPA) source. For the laser ablation, a low-priced 445-nm continuous-wave diode laser pointer, with a power of 1 W, was used. The combination of the simple, low-budget laser pointer and the FAPA ion source has made this experimental arrangement broadly available, also for small laboratories. The approach was successfully applied for the characterization of low molecular weight compounds separated on TLC plates, such as a mixture of pyrazole derivatives, alkaloids (nicotine and sparteine), and an extract from a drug tablet consisting of paracetamol, propyphenazone, and caffeine. The laser pointer used was capable of ablating organic compounds without the need of application of any additional substances (matrices, staining, etc.) on the TLC spots. The detection limit of the proposed method was estimated to be 35 ng/cm^2^ of a pyrazole derivative.

**Graphical abstract:**
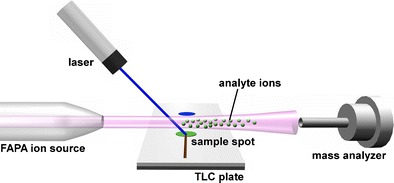
Schematic illustration of new TLC-FAPA setup with diode laser ablation

Schematic illustration of new TLC-FAPA setup with diode laser ablation

## Introduction

Ambient mass spectrometry allows for the recording of mass spectra of analytes directly from their natural mixtures [1]. This technique requires no or only minimal sample preparation and can be operated at room temperature under atmospheric pressure. This feature allows for the analysis and identification of unstable samples, including in vivo experiments. The most commonly applied ionization methods operating at ambient conditions and linked to mass spectrometry are desorption electrospray ionization (DESI) [2, 3], direct analysis in real time (DART) [4], atmospheric pressure chemical ionization (APCI) [5], dielectric barrier discharge ionization (DBDI) [6–8], electrospray-assisted laser desorption/ionization (ELDI) [9], and flowing atmospheric pressure afterglow (FAPA) [10–13]. During analysis of bulk materials, these methods are capable of analyzing compounds that are present on the surface of the sample. Therefore, they are ideal for fast identification of analytes that have previously been deposited on different types of surfaces.

The main features of thin-layer chromatography (TLC) are low cost, easy operation under ambient conditions, and the need of only small amounts of solvent [1]. Moreover, for each separation, a new plate is used; therefore, there is no “memory effect” that may occur in other chromatographic techniques. For the same reason, TLC plates may be applied for the analysis of unknown samples, for which there is a risk that they can damage much more expensive liquid chromatography or gas chromatography columns. Commercially available TLC plates can be obtained with stationary phases made of silica, modified (e.g., reversed-phase, CN or diol-modified) silica, cellulose, or aluminum oxide, thus allowing for the separation of various types of compounds. To obtain additional information about the structure of separated compounds, other techniques must be applied. Using UV light or appropriate TLC visualization reagents allows only for the moderate determination of the optical or chemical properties of separated components. To improve the quality of information that can be obtained after TLC analysis, several approaches combining TLC and mass spectrometry (MS) have been presented [1, 14–18].

Thin-layer chromatography-mass spectrometry (TLC-MS) techniques rely on MS analysis of the spots that have been obtained after development of a TLC plate. Recently, many direct-sampling TLC-MS techniques, which allow obtaining mass spectra of the analytes by desorption and subsequent ionization directly from the TLC plate, such as TLC-matrix-assisted laser desorption/ionization (TLC-MALDI) [19], TLC-desorption electrospray ionization (TLC-DESI) [20], TLC-electrospray-assisted laser desorption/ionization (TLC-ELDI) [21], TLC-easy ambient sonic spray ionization (TLC-EASI) [22], TLC-direct analysis in real time (TLC-DART) [23], and TLC-atmospheric-pressure chemical ionization (TLC-APCI) [24] have been developed. Our group has recently presented the TLC-dielectric barrier discharge ionization (TLC-DBDI) setup and successively used it for characterization of small organic compounds separated on TLC plates with thermal desorption [25]. TLC-MS setups have been applied for identification of fatty acids [26, 27], synthesis products [28], alkaloids [29], and drugs [30].

In this study, we present a combination of FAPA ionization with the continuous-wave diode laser (445 nm) ablation for MS characterization of various compounds resolved on TLC plates. The Combination of laser irradiation and ambient mass spectrometry has been used for characterization of organic compounds separated on TLC plates [21, 24, 31–35]. The overall cost of this system, particularly a scientific laser module and an ambient ion source, is usually very high, which makes such arrangements unfavorable in comparison with the commercially available TLC-MS setups, such as the CAMAG TLC-MS interface. The combination described in this work is attractive due to a very low cost of this type of laser and simple construction of the FAPA ion source.

## Materials and methods

### Chemicals and reagents

All reagents used were commercial products. Ethyl 2,4-dioxovalerate, hydrazine hydrate, 2-hydrazinopyridine, 2-hydrazinobenzothiazole, lithium aluminum hydride, nicotine, sparteine, and anhydrous tetrahydrofuran (THF) were obtained from Sigma-Aldrich (St. Louis, MO, USA). Anhydrous ethyl alcohol and all other solvents were of p.a. grade, obtained from Avantor Performance Materials Poland S.A. (Gliwice, Poland) and were used without further purification. The commonly used analgesic, consisting of paracetamol, propyphenazone, and caffeine, was obtained from the local pharmacy.

Synthesis of pyrazole derivatives used in this work as analytes for the TLC-FAPA technique has been described in detail in our previous publication [25]. For all TLC-FAPA experiments, the Merck Millipore TLC silica gel 60 F_254_ aluminum sheets was applied.

### TLC preparation

Five pyrazole derivatives were dissolved in dichloromethane to obtain one solution containing 10 mg mL^−1^ of each derivative. One microliter of the prepared mixture was deposited on a TLC plate, which was subsequently developed with CH_2_Cl_2_/Et_2_O (10:1, *v*/*v*).

A mixture of nicotine and sparteine was prepared by dissolving these compounds in methanol to obtain 10 mg mL^−1^ solution of each of these compounds. One microliter of the prepared mixture was spotted on a TLC plate, which was subsequently developed with CH_2_Cl_2_/MeOH (5:1, *v*/*v*).

One tablet of the commonly used analgesic consisting of paracetamol (250 mg), propyphenazone (150 mg), and caffeine (50 mg) was ground with a mortar and pestle, added to 10 mL of methanol, and filtered. One microliter of the prepared solution was spotted on a TLC plate, which was subsequently developed with CH_2_Cl_2_/MeOH (10:1, *v*/*v*).

### Instrumentation

A NOVA011 (ERTEC, Wroclaw, Poland) FAPA ambient plasma source was used for the ionization of the compounds thermally desorbed by diode laser. Briefly, the main part of the source is a quartz tube from the rear side that is axially mounted along a Teflon head, which also serves as a compartment for the cable delivering high-voltage potential to the anode and as a housing for the helium gas fitting. The discharge power is adjustable within the range of 3–30 W. The open circuit voltage (OCV) is in excess of 20 kV, which always assures self-ignition of the helium discharge. The helium discharge gas was continuously delivered at a flow rate of 1.0 L min^−1^. More technical details describing this ion source can be found in our previous publication [13].

The laser used for ablation of organic compounds from TLC plates was a continuous-wave 445-nm diode laser pointer with an output power of ca. 1 W and a laser diode manufactured by Nichia Corporation (Anan, Tokushima, Japan), and was powered by a 18650 lithium ion cell. It was bought over the Internet for ca. 150 € from a supplier that specializes in selling laser pointers.

A Bruker Esquire 3000 quadrupole ion trap mass spectrometer (Bruker Daltonics, Bremen, Germany) was applied for all measurements. The standard ESI source settings were also found to be optimal for the FAPA source, with the exception of the capillary voltage where a much lower potential (1 kV) than in the standard ESI settings (4.5 kV) was used [13].

### TLC-FAPA measurements

**Fig. 1:**
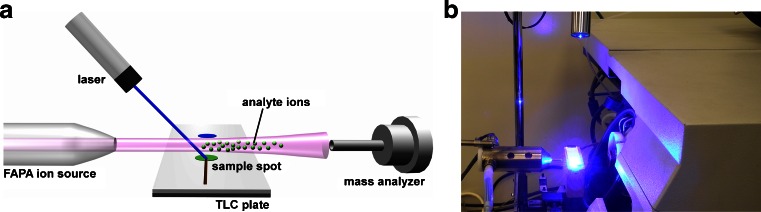
Schematic illustration (**a**) and photograph (**b**) of new TLC-FAPA setup with diode laser ablation

**Fig. 2:**
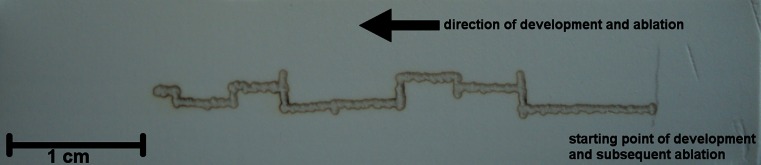
Photograph of the ablated TLC plate

Figure 1 shows a photograph and a scheme of the experimental assembly consisting of the mass spectrometer, a FAPA ion source, the laser, and the TLC plate mounted on the *x-y* stage. The FAPA ion source was positioned on the axis of the inlet of the mass spectrometer with the tip located 50 mm from the MS heated capillary. The TLC plate was placed 10 mm below the plasma stream. The laser was positioned ca. 10 cm above the surface of the TLC plate, and the laser beam was focused on the plate’s surface. During analysis, the plate was manually moved using an *x-y* manipulator to scan the surface. To allow ablation, the TLC plate has to be hit by a laser, and its effectiveness was observed as the change in color of the stationary phase from white to brown. To initiate this process, the laser beam must be focused on the plate to obtain the spot with a diameter of ca. 0.8 mm. Unfortunately, white background of the plate causes the laser beam to dissipate. Therefore, to avoid energy loss, a thin line was drawn on the plate with a pencil to allow the laser spot to initiate ablation from the graphite mark. When the laser spot had passed this line, the ablation process was spontaneously continued, which was observed as a dark laser track. The darkening of the TLC surface initiated further TLC ablation, leading to the formation of a path on the plate (a photograph of the analyzed TLC plate is presented in Fig. 2). The change in the color of the TLC plate surface, either by a pencil or laser beam, was essential to initiate ablation because laser energy was no longer dissipated. Without the ablation process, no mass spectra could be obtained. The spatial resolution of the proposed method is therefore limited only by the diameter of the laser beam because ablation occurs selectively at the place of irradiation. It was established that this change in color of the TLC surface is observed when the temperature rises above 500 °C (measured by a pyrometer). The desorbed molecules, as vapors, were introduced into the plasma stream, where they were ionized by FAPA, and subsequently migrated to the inlet of the mass spectrometer. The time required to obtain mass spectra from the moment of irradiation of the TLC spot was less than 3 s.

### Safety hazard note

The employed laser was of safety class 4, which means that it can burn the skin or cause permanent eye damage, as a result of direct, diffuse, or indirect beam viewing. Working with this class of lasers requires wearing certified safety glasses or protection goggles. Another issue that should be taken into consideration is an effective hood, to collect exhaust of all vapors, sometimes toxic, generated by ablation.

## Results and discussion

Ambient mass spectrometry becomes very popular due to the fast analysis time and negligible or short sample preparation. However, this technique often does not transfer a sufficient amount of energy to the sample to cause desorption and ionization; therefore, additional tools must be used. The setup proposed by our group and described in the experimental section is of a low price because it consists of a simple FAPA construction and a low-budget laser pointer. The laser pointer was utilized because plasma produced by the FAPA source does not have adequate energy to cause desorption of the compounds on the TLC surface. Below, the applications of the TLC-MS setup will be illustrated.

### Analysis of pyrazole derivatives

**Fig. 3:**
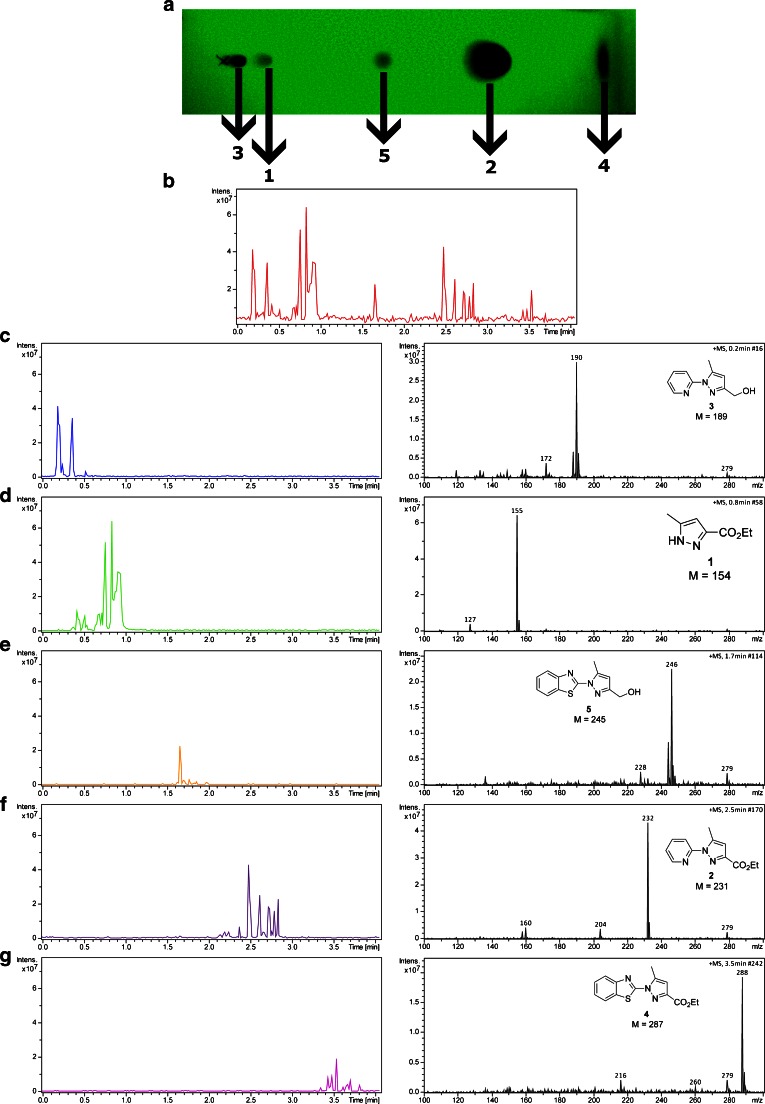
Photograph of the TLC plate (**a**) and BPC obtained during analysis of a mixture of compounds **1**–**5** separated on a TLC plate (**b**). EIC along with mass spectra of compounds: **3** (**c**), **1** (**d**), **5** (**e**), **2** (**f**), and **4** (**g**)

Figure 3a shows a photograph of the developed TLC plate at 254 nm. Values of *R*_*f*_ factor are 0.05, 0.14, 0.41, 0.71, and 0.96 for compounds **3**, **1**, **5**, **2**, and **4**, respectively. Figure 3b shows base peak chromatogram (BPC) obtained during manual scanning of the TLC plate at constant speed, indicating that the mixture of all five pyrazole derivatives has been resolved. Panels c to g of Fig. 3 show the extracted ion chromatograms (EIC) of each derivative, along with their mass spectra and structures. The intensity of the signals of particular ions across each spot varies, as can be observed during analysis of all compounds examined in this work. When a signal of the analyte was detected, the spot was thoroughly scanned. The vapors released from the spot were introduced into the mass spectrometer. The observed intensity “bursts” visible in BPC and EIC arise from the uneven distribution of the analytes across the surface of the TLC plate. This method of analysis does not allow obtaining a well-defined TLC “map,” on which the distance/mass signal correlation can be presented but is dedicated for a rapid, manual screening of the spots to minimize analysis time and provide unambiguous identification of separated molecules. All peaks present in the BPC can be found in the EIC, which means that during TLC scanning, background ions have constant intensity (no background correction has been used). The most intense background ion is visible at *m*/*z* 279, corresponding to di-*n*-butyl phthalate (DBP), a plasticizer leaking from the tubing used in our setup. The mixture of five pyrazole derivatives has been used as an example to show proof of concept, because these compounds are well resolved on the TLC plate and, in our previous experiments [25], all these compounds were observed as protonated molecules on the mass spectra. The smallest difference in *R*_*f*_ factor equal to 0.09 is between compounds **3** and **1,** which can be clearly seen in the BPC. Only minor decomposition of the compounds caused by laser irradiation or pyrolysis has been observed. Alcohols, particularly compounds **3** and **5**, show the most intense fragment ions derived from the loss of water molecule at *m*/z 172 and *m*/z 227, respectively. Compound **1** shows one fragment ion at *m*/*z* 127 generated after elimination of the ethyl group. Esters **2** and **4** show fragment ions resulting from the loss of the ethyl group at *m*/*z* 204 and *m*/*z* 260, and after the loss of the ethyl group and decarboxylation at *m*/*z* 160 and *m*/*z* 216, respectively. Nevertheless, each spectrum represents the most intense ion corresponding to the protonated molecule of pyrazole derivatives.

### Analysis of nicotine and sparteine

**Fig. 4:**
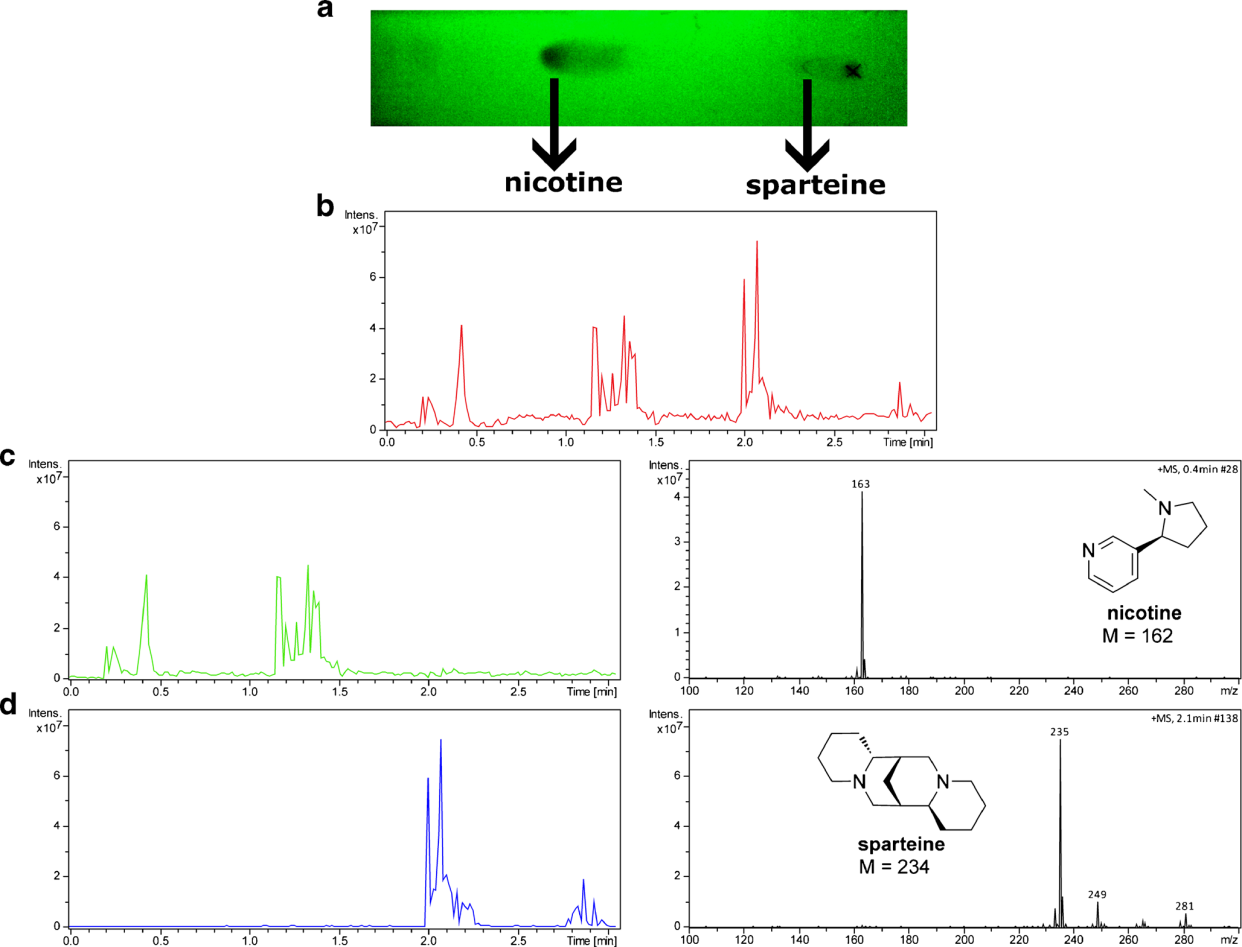
Photograph of the TLC plate (**a**) and BPC obtained during analysis of a mixture of nicotine and sparteine separated on a TLC plate (**b**). EIC along with the mass spectra of nicotine (**c**) and sparteine (**d**)

Figure 4 shows a photograph of a developed TLC plate at 254 nm, and also BPC and EIC obtained during manual scanning of the TLC plate, on which a mixture of nicotine (*R*_*f*_ = 0.61) and sparteine (*R*_*f*_ = 0.11) has been resolved. During this analysis, each spot on a TLC plate was scanned two times at distinct regions. Moreover, it is worth noting that the diameter of a laser beam is much smaller than the size of the spots which increases the resolution of the analyses as compared to, e.g., thermal desorption on the TLC plate. Therefore, multiple, good-quality mass spectra can be obtained and averaged during one manual scanning. No decomposition of the compounds caused by laser irradiation or pyrolysis has been observed, and both alkaloids are presented on the mass spectra as protonated molecules.

### Analysis of a drug tablet

**Fig. 5:**
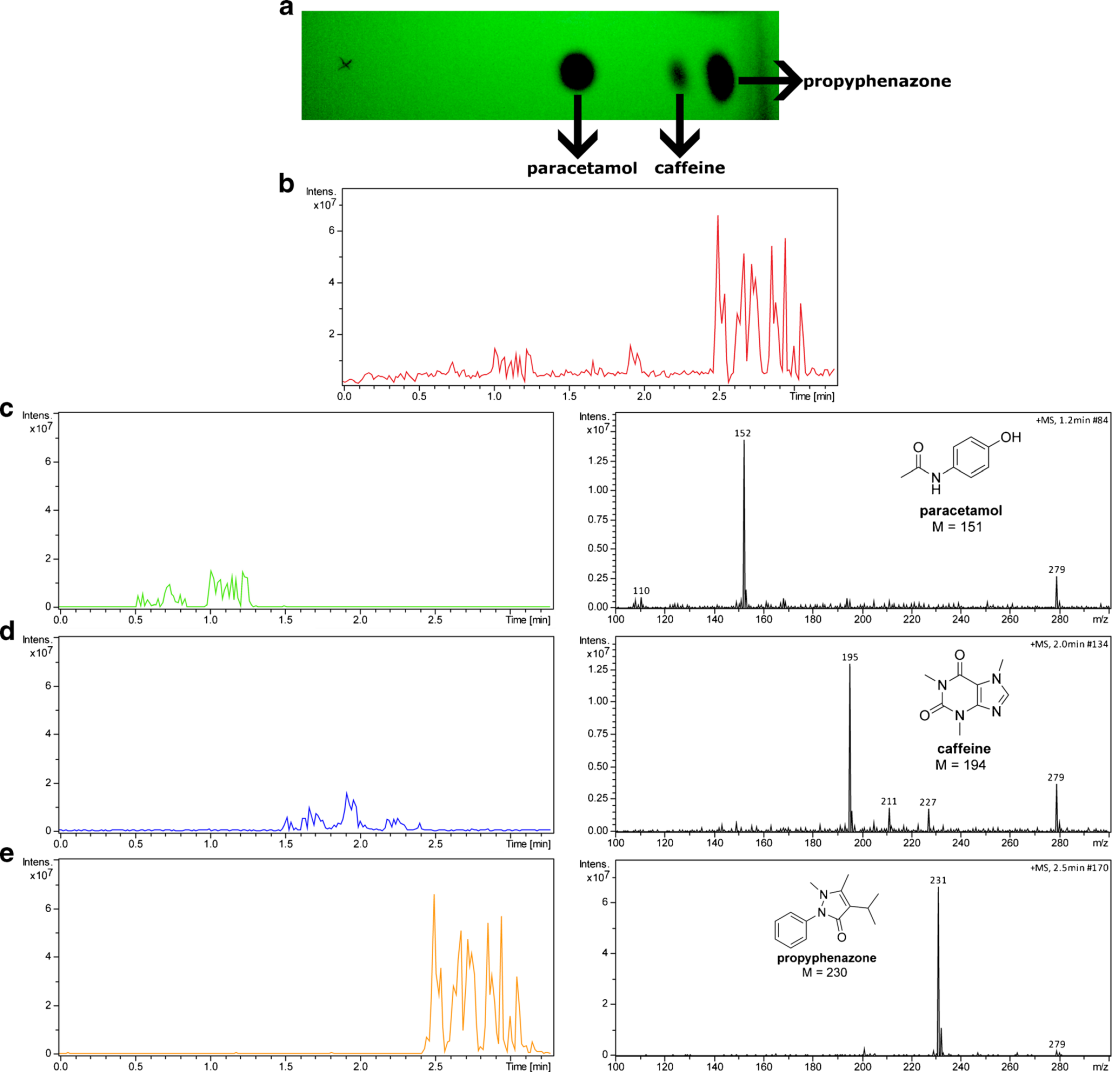
Photograph of the TLC plate (**a**) and BPC obtained during analysis of an extract of a drug tablet containing paracetamol, propyphenazone, and caffeine separated on a TLC plate (**b**). EIC along with mass spectra of paracetamol (**c**), caffeine (**d**), and propyphenazone (**e**)

Figure 5 shows a photograph of a developed TLC plate at 254 nm, and BPC and EIC obtained during manual scanning of the TLC plate, on which methanolic extract from a tablet of the commonly used analgesic containing paracetamol (*R*_*f*_ = 0.59), caffeine (*R*_*f*_ = 0.80), and propyphenazone (*R*_*f*_ = 0.90) has been resolved. All components are visible on the mass spectra as major ions corresponding to the protonated molecules. Slight fragmentation of paracetamol has been observed during analysis. Paracetamol shows a fragment ion resulting from the loss of the acetyl group at *m*/*z* 110. The intensity of an ion belonging to propyphenazone on the total ion chromatogram is around five times higher than that of paracetamol or caffeine, despite the fact that the concentration of propyphenazone (150 mg in the drug tablet) in the extract is lower than the paracetamol content (250 mg in the drug tablet). This observation can be explained by the differences in ionization or desorption efficiencies of these compounds.

### Detection limit

**Fig. 6:**
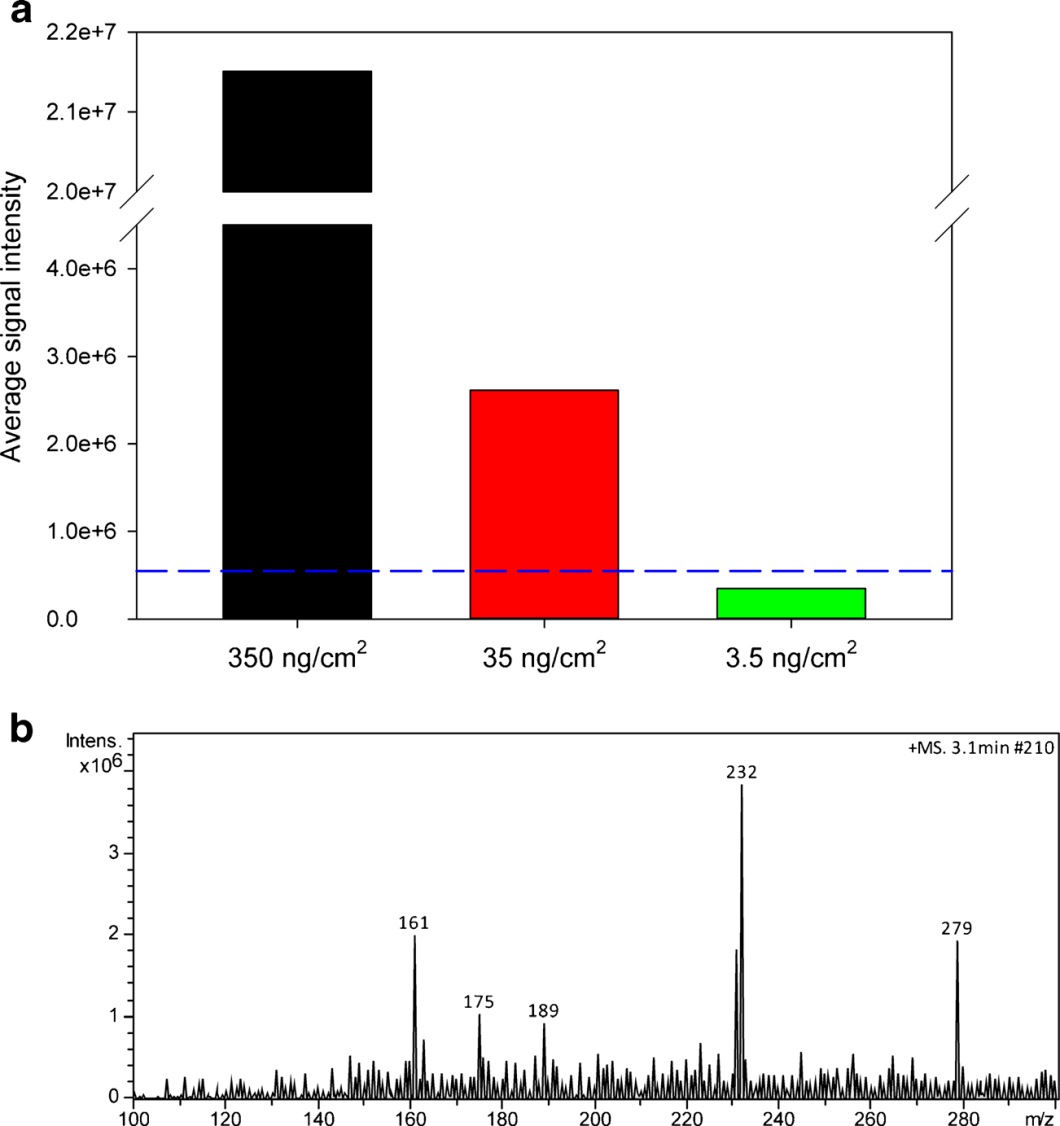
Results of LOD experiments (**a**); the LOD value (represented by the *blue dashed line*) was calculated as an average signal intensity for the reagent blank plus three times the standard deviation of the reagent blank’s signal. Mass spectrum of compound **2** at concentration of 35 ng/cm^2^ (**b**)

To estimate the detection limit of the method, TLC-MS analysis of pyrazole derivative **2** of various amounts per square centimeter has been performed. Compound **2** has been selected because the intensity of its signals during TLC-MS analysis was moderate, which means that among all analytes examined, its ablation/ionization properties were around average. The results of the limit of detection (LOD) experiments are presented in Fig. 6. In all experiments, the average intensities of a signal at *m*/*z* 232 were calculated from the EIC after 10 measurements and the relative standard deviations (RSDs) were below 22 %. In accordance with the LOD definition (LOD = mean blank value + 3 × standard deviation) [36, 37], if the average intensity of the signal at *m*/*z* 232 exceeds the LOD value, then the analyte is within detection range. The mean blank value of a signal at *m*/*z* 232 was obtained in the absence of compound **2** on the TLC surface. Afterwards, the standard deviation of 10 measurements has been calculated. The LOD value was marked in Fig. 6 by the blue dashed line. For the TLC-FAPA procedure, the detection limit was estimated at 35 ng/cm^2^ (10 ng per spot). Within the concentration from 3.5 to 350 ng/cm^2^, the analytical response was linear with a correlation coefficient *R*^2^ of 0.91. Figure 6b shows the mass spectrum obtained for 35 ng/cm^2^ of substance on a TLC plate. A signal at *m*/*z* 232 has the highest intensity; however, it is only ca. three times higher than the intensity of background ions and ca. two times higher than the intensity of the DBP contaminant. At a concentration of 35 ng/cm^2^, the compound could not be observed under UV light. The result of the detection limit is determined by many factors, such as type of analyte, thickness, and composition of silica gel, laser power, and positioning of a TLC plate, which can affect signal intensity. The estimated detection limit corresponds to detection limits established for other ambient TLC-MS setups, for instance ca. 25 ng per spot [38] and 2–23 ng per spot [39] obtained for TLC-DART-MS, ca. 5 ng per spot (size ranging from 3 to 5 mm) obtained for TLC/DESI-MS [40], and ca. 1 ng per spot obtained for TLC-ELDI-MS [21].

## Conclusions

Flowing atmospheric pressure afterglow has been successfully coupled with laser ablation to obtain mass spectra of the compounds resolved on TLC plates. Different types of analytes, particularly synthetic pyrazole derivatives, natural compounds (alkaloids), and active components of the drug tablet were resolved and subsequently analyzed with this simple TLC-MS setup. The diode laser pointer used in this work was capable of ablating organic compounds without the need of application of any additional substances on the spots, such as graphite or matrices, contrary to other procedures [41]. We have shown that TLC-FAPA is very fast and sensitive for the on-the-spot detection and characterization of compounds directly evaporated from TLC plates, and also shows a much higher resolution of the analyses, as the laser beam can be focused. The major advantage of the presented approach is its ability to analyze samples of various polarities, low cost, good resolution, and fast analysis time. Moreover, FAPA involving ambient plasma can also be constructed from the low-cost components. We believe that this new TLC-MS setup has a good application potential as a cheap, stand-alone module that can easily be attached to different types of mass spectrometers, can also be miniaturized, and may also be competitive to the commercially available TLC-MS setups.

## References

[1] Cheng S-C, Huang M-Z, Shiea J (2011). Thin layer chromatography/mass spectrometry. J Chromatogr A.

[2] Takáts Z, Wiseman JM, Gologan B, Cooks RG (2004). Mass spectrometry sampling under ambient conditions with desorption electrospray ionization. Science.

[3] Cooks RG, Ouyang Z, Takats Z, Wiseman JM (2006). Ambient mass spectrometry. Science.

[4] Cody RB, Laramée JA, Durst HD (2005). Versatile new ion source for the analysis of materials in open air under ambient conditions. Anal Chem.

[5] Carroll DI, Dzidic I, Stillwell RN, Horning MG, Horning EC (1974). Subpicogram detection system for gas phase analysis based upon atmospheric pressure ionization (API) mass spectrometry. Anal Chem.

[6] Na N, Zhao M, Zhang S, Yang C, Zhang X (2007). Development of a dielectric barrier discharge ion source for ambient mass spectrometry. J Am Soc Mass Spectrom.

[7] Mielczarek P, Smoluch M, Kotlinska JH, Labuz K, Gotszalk T, Babij M, Suder P, Silberring J (2015). Electrochemical generation of selegiline metabolites coupled to mass spectrometry. J Chromatogr A.

[8] Smoluch M, Ceglowski M, Kurczewska J, Babij M, Gotszalk T, Silberring J, Schroeder G (2014). Molecular scavengers as carriers of analytes for mass spectrometry identification. Anal Chem.

[9] Shiea J, Huang M-Z, Hsu H-J, Lee C-Y, Yuan C-H, Beech I, Sunner J (2005). Electrospray-assisted laser desorption/ionization mass spectrometry for direct ambient analysis of solids. Rapid Commun Mass Spectrom.

[10] Andrade FJ, Shelley JT, Wetzel WC, Webb MR, Gamez G, Ray SJ, Hieftje GM (2008). Atmospheric pressure chemical ionization source. 2. Desorption–ionization for the direct analysis of solid compounds. Anal Chem.

[11] Smoluch M, Silberring J, Reszke E, Kuc J, Grochowalski A (2014). Determination of hexabromocyclododecane by flowing atmospheric pressure afterglow mass spectrometry. Talanta.

[12] Smoluch M, Mielczarek P, Reszke E, Hieftje GM, Silberring J (2014). Determination of psychostimulants and their metabolites by electrochemistry linked on-line to flowing atmospheric pressure afterglow mass spectrometry. Analyst.

[13] Smoluch M, Reszke E, Ramsza A, Labuz K, Silberring J (2012). Direct analysis of methcathinone from crude reaction mixture by flowing atmospheric-pressure afterglow mass spectrometry. Rapid Commun Mass Spectrom.

[14] Sherma J (2008). Planar chromatography. Anal Chem.

[15] Somsen GW, Morden W, Wilson ID (1995). Planar chromatography coupled with spectroscopic techniques. J Chromatogr A.

[16] Wilson ID (1999). The state of the art in thin-layer chromatography–mass spectrometry: a critical appraisal. J Chromatogr A.

[17] Morlock G, Schwack W (2010). Coupling of planar chromatography to mass spectrometry. Trends Anal Chem.

[18] Sherma J (2010). Planar chromatography. Anal Chem.

[19] Gusev AI, Proctor A, Rabinovich YI, Hercules DM (1995). Thin-layer chromatography combined with matrix-assisted laser desorption/ionization mass spectrometry. Anal Chem.

[20] Van Berkel GJ, Ford MJ, Deibel MA (2005). Thin-layer chromatography and mass spectrometry coupled using desorption electrospray ionization. Anal Chem.

[21] Lin S-Y, Huang M-Z, Chang H-C, Shiea J (2007). Using electrospray-assisted laser desorption/ionization mass spectrometry to characterize organic compounds separated on thin-layer chromatography plates. Anal Chem.

[22] Haddad R, Milagre HMS, Catharino RR, Eberlin MN (2008). Easy ambient sonic-spray ionization mass spectrometry combined with thin-layer chromatography. Anal Chem.

[23] Smith NJ, Domin MA, Scott LT (2008). HRMS directly from TLC slides. A powerful tool for rapid analysis of organic mixtures. Org Lett.

[24] Herdering C, Reifschneider O, Wehe CA, Sperling M, Karst U (2013). Ambient molecular imaging by laser ablation atmospheric pressure chemical ionization mass spectrometry. Rapid Commun Mass Spectrom.

[25] Cegłowski M, Smoluch M, Babij M, Gotszalk T, Silberring J, Schroeder G (2014). Dielectric barrier discharge ionization in characterization of organic compounds separated on thin-layer chromatography plates. PLoS One.

[26] Mirabelli M, Coviello G, Volmer D (2015). Determining fatty acids by desorption/ionization mass spectrometry using thin-layer chromatography substrates. Anal Bioanal Chem.

[27] Alberici L, Oliveira HF, Catharino R, Vercesi A, Eberlin M, Alberici R (2011). Distinct hepatic lipid profile of hypertriglyceridemic mice determined by easy ambient sonic-spray ionization mass spectrometry. Anal Bioanal Chem.

[28] Himmelsbach M, Waser M, Klampfl C (2014). Thin layer chromatography–spray mass spectrometry: a method for easy identification of synthesis products and UV filters from TLC aluminum foils. Anal Bioanal Chem.

[29] Kukula-Koch W, Mroczek T (2015). Application of hydrostatic CCC–TLC–HPLC–ESI-TOF-MS for the bioguided fractionation of anticholinesterase alkaloids from Argemone mexicana L. roots. Anal Bioanal Chem.

[30] Kuwayama K, Tsujikawa K, Miyaguchi H, Kanamori T, Iwata Y, Inoue H (2012). Rapid, simple, and highly sensitive analysis of drugs in biological samples using thin-layer chromatography coupled with matrix-assisted laser desorption/ionization mass spectrometry. Anal Bioanal Chem.

[31] Zhang J, Zhou Z, Yang J, Zhang W, Bai Y, Liu H (2011). Thin layer chromatography/plasma assisted multiwavelength laser desorption ionization mass spectrometry for facile separation and selective identification of low molecular weight compounds. Anal Chem.

[32] Cheng S-C, Huang M-Z, Shiea J (2009). Thin-layer chromatography/laser-induced acoustic desorption/electrospray ionization mass spectrometry. Anal Chem.

[33] Peng S, Edler M, Ahlmann N, Hoffmann T, Franzke J (2005). A new interface to couple thin-layer chromatography with laser desorption/atmospheric pressure chemical ionization mass spectrometry for plate scanning. Rapid Commun Mass Spectrom.

[34] Cheng S-C, Huang M-Z, Wu L-C, Chou C-C, Cheng C-N, Jhang S-S, Shiea J (2012). Building blocks for the development of an interface for high-throughput thin layer chromatography/ambient mass spectrometric analysis: a green methodology. Anal Chem.

[35] Salo PK, Vilmunen S, Salomies H, Ketola RA, Kostiainen R (2007). Two-dimensional ultra-thin-layer chromatography and atmospheric pressure matrix-assisted laser desorption/ionization mass spectrometry in bioanalysis. Anal Chem.

[36] MacDougall D, Crummett WB (1980). Guidelines for data acquisition and data quality evaluation in environmental chemistry. Anal Chem.

[37] Ceglowski M, Kurczewska J, Smoluch M, Reszke E, Silberring J, Schroeder G (2015). Magnetic scavengers as carriers of analytes for flowing atmospheric pressure afterglow mass spectrometry (FAPA-MS). Analyst.

[38] Kim HJ, Oh MS, Hong J, Jang YP (2011). Quantitative analysis of major dibenzocyclooctane lignans in Schisandrae fructus by online TLC-DART-MS. Phytochem Anal.

[39] Kiguchi O, Oka K, Tamada M, Kobayashi T, Onodera J (2014). Thin-layer chromatography/direct analysis in real time time-of-flight mass spectrometry and isotope dilution to analyze organophosphorus insecticides in fatty foods. J Chromatogr A.

[40] Van Berkel GJ, Tomkins BA, Kertesz V (2007). Thin-layer chromatography/desorption electrospray ionization mass spectrometry: investigation of goldenseal alkaloids. Anal Chem.

[41] Peng S, Ahlmann N, Kunze K, Nigge W, Edler M, Hoffmann T, Franzke J (2004). Thin-layer chromatography combined with diode laser desorption/atmospheric pressure chemical ionization mass spectrometry. Rapid Commun Mass Spectrom.

